# Assessment of the quality of life, muscle strength, and dynamic balance of elderly Kendo players

**DOI:** 10.6061/clinics/2017(11)03

**Published:** 2017-11

**Authors:** Dário Lucas Costa de Mendonça, Angelica Castilho Alonso, Júlia Maria D’Andrea Greve, Luiz Eugênio Garcez-Leme

**Affiliations:** I Laboratorio de Estudo do Movimento, Laboratório de Investigação Médica do Sistema Músculo-Esquelético LIM 41, Instituto de Ortopedia e Traumatologia, Hospital das Clínicas (HCFMUSP), Faculdade de Medicina, Universidade de Sao Paulo, Sao Paulo, SP, BR; II Grupo de Ortogeriatria, Instituto de Ortopedia e Traumatologia, Hospital das Clínicas (HCFMUSP), Faculdade de Medicina, Universidade de Sao Paulo, Sao Paulo, SP, BR; III Programa de Mestrado em Ciencias do Envelhecimento, Universidade Sao Judas Tadeu, Sao Paulo, SP, BR

**Keywords:** Aged, Martial Arts, Muscle Strength, Postural Balance, Quality of Life

## Abstract

**OBJECTIVE:**

To compare Kendo players with active elderly adults in terms of quality of life, functional aspects (muscle strength, postural balance) and body composition.

**METHODS:**

This was a controlled cross-sectional study. Twenty elderly individuals were divided into two groups: the Kendo group, with an average age of 71.8 (5.4) years, and the Control group, with an average age of 73.1 (4.8) years. Quality of life was evaluated using a questionnaire (WHOQOL-bref and WHOQOL-old); body composition was evaluated with a bioimpedance scale (InBody230); hand-grip strength was assessed with a portable manual dynamometer (Jamar SH 5001); flexor and extensor muscle strength of the knees was evaluated with an isokinetic device (Biodex® System 3 model Biodex Multi Joint System, BIODEX); and dynamic balance was assessed using a force platform (Balance Master System, Neurocom International, Inc.,® Clackamas County, Oregon, USA).

**RESULTS:**

The groups were statistically homogeneous in terms of socio-demographic characterization, body composition, muscle strength, and dynamic balance, but the Control group was faster in the sit-to-stand test (*p*=0.03). The Kendo group had a statistically significantly better quality of life; in the WHOQOL-bref, these differences were present in the physical (*p*≤0.001) and environment (*p*=0.004) domains, and in the WHOQOL-old, these differences were present in social participation (*p*=0.001) and in past, present, and future activities (*p*=0.019).

**CONCLUSION:**

The results suggest that Kendo is a health-promoting activity that improves the quality of life, functional aspects (muscle strength and postural balance) and body composition of players.

## INTRODUCTION

With increased life expectancy, there is a growing interest in the study of quality of life (QoL) with the aim of recognizing healthy populations and highlighting their health-promoting practices. Therefore, it is important to identify cultural groups to study the strategies they have adopted and to disclose different ways of ageing well 1. From this perspective, the improved QoL of the population must be considered a primary objective of our activities as scientists and clinicians 2.

QoL has been defined by the World Health Organization as “the individuals’ perception of their position in life in the context of the culture and value systems in which they live and in relation to their goals, expectations, standards, and concerns” 3. To improve the QoL and life expectancy of the elderly, regular physical activity is recommended 4.

Kendo is a martial art that has its origin in kenjutsu (samurai fencing) and seeks to shape the mind, body, and spirit through correct training in sword handling 5.

The cultural and social aspects of Kendo have been studied in depth, but its impact on health and QoL is unknown. To promote adherence to physical activity programs, such activities should accommodate the participant’s lifestyle, have cultural context and respect cultural traditions to improve the participant’s values and meet his or her needs 6.

For this reason, the objective of this study was to compare Kendo players with active elderly adults in terms of QoL, functional aspects (muscle strength and postural balance) and body composition.

## MATERIALS AND METHODS

### Study Type and Ethics

This was a controlled cross-sectional study conducted at the Motion Study Laboratory of the Institute of Orthopaedics and Traumatology, Clinical Hospital, University of São Paulo School of Medicine. It was approved by the Ethics Committee of the School of Medicine of the University of Sao Paulo (Universidade de São Paulo- USP) under N. 012/14.

### Description of the Sample

A convenience sample of twenty elderly adults divided into two groups paired by age were assessed: the Kendo group, comprising 10 players, and the Control group, comprising 10 physically active elderly adults that did not practice Kendo. All the participants underwent a QoL evaluation. For the functional evaluation, two players did not attend because they lived far away from the laboratory, but this fact did not interfere with the primary outcomes of the study.

The inclusion criteria for both groups were as follows: male volunteers aged ≥60 years who were autonomous and had a positive self-perception of their health, regardless of comorbidities; absence of vestibular, proprioceptive, auditory, or neurological impairment and/or any mental disturbances or disorders; no use of medications that might compromise postural balance or that interfere with musculoskeletal metabolism; absence of lesions, surgery, or disease over the previous six months that might cause lower-limb joint limitations; absence of any lower-limb dysmetria; presence of clinically normal gait without claudication. For the Kendo group, more than five years of practicing the martial art was required. For the Control group, participants were required to have no involvement with Kendo and to participate regularly in physical activities.

The exclusion criteria were an inability to understand and respond to the applied questionnaires or a request to leave the study.

Physical activity level was classified according to the brief IPAQ 7. In the Kendo group, five participants were classified as very active (50%) and five as active (50%); in the Control group, four participants were classified as very active (40%) and six as active (60%).

In the Kendo group, eight participants (80%) practiced twice a week, one participant (10%) practiced three times a week, and one participant (10%) practiced five times a week. Iaidô (withdrawal-of-sword technique) was practiced by four participants (40%) once a week. All the Kendo players had practiced for more than five years and were ranked at the Dan level: one player (10%) was ranked as 1st Dan, one (10%) as 2nd Dan, three (30%) as 4th Dan, and five (50%) as 7th Dan. Two participants (20%) exercised with a resistive load twice a week. One participant (10%) participated in aqua aerobics twice a week. One participant (10%) engaged in vigorous walking five times a week.

In the Control group, eight participants (80%) performed vigorous walking, as follows: three participants (30%) walked seven times a week, three (30%) walked four times a week, and one (10%) walked twice a week and ran once a week. Eight participants (80%) exercised with a resistive load: three participants (30%) did so once a week, three (30%) did so twice a week, one (10%) did so three times a week, and one (10%) did so five times a week. One participant (10%) participated in aqua aerobics twice a week. Two participants (20%) practiced yoga once a week. Two participants (20%) practiced Tai Chi Chuan once a week.

### Procedures

The elderly players were invited to participate in the Kendo group during Kendo events at Sao Paulo State. The elderly physically active individuals were invited to participate in the Control group at the University for the Third Age of the University of Sao Paulo (Universidade de São Paulo–USP). The groups were paired by age, and all subjects signed an Informed Consent Form.

### Quality of Life Assessment

The QoL assessment was conducted with the WHOQOL-bref and WHOQOL-old questionnaires. The participants self-administered the questionnaires to avoid embarrassment and influencing responses 8. The WHOQOL scores were calculated on a computer using Excel 9.

### Anthropometric Assessment and Body Composition

Height in centimetres was measured with a stadiometer. For this measurement, the distance between the platform of the stadiometer and the vertex of the head was considered, using the Frankfurt plane as the basis. Body mass in kilograms, skeletal muscle mass, and body mass index (BMI) were measured using an InBody230 bioimpedance scale.

### Muscle Strength Assessment

#### Two assessment protocols were used

1) Hand-grip strength: A manual Jamar® dynamometer was used with a measurement adopted by the American Society of Hand Therapists (ASHT). The hands were switched after each manoeuvre, with one minute of rest between tests; the first manoeuvre was performed with the dominant limb. The first two manoeuvres were performed to familiarize the participant with the test, and then three additional manoeuvres were performed with each hand. For analysis purposes, the average of the last three measurements for each limb was calculated 10.

2) Knee extension and flexion strength: A Biodex® multi-joint System3 isokinetic dynamometer (Biodex Medical Systems, Inc., Shirley, NY, USA) was used. Concentric movements were chosen (knee flexion and extension) starting at 90° of flexion and proceeding to 20° of extension and were corrected by the gravitational force at half amplitude according to the manufacturer’s instructions. The angular velocity of choice was 60°/second because it allows the recruitment of a greater number of motor units for rapid muscle contraction strength. To familiarize the participants with the device, four submaximal repetitions were performed, and each was followed by an interval of 60 seconds. For the test, two series of five repetitions without interruption were performed for knee extension and flexion. Between the series, there was an interval of 60 seconds. The test started with the dominant limb, and after 60 seconds, the test was performed with the non-dominant limb. For data analysis, the values of the second series of each measured limb were used, taking into consideration the effects of motor familiarity related to repetition 11.

#### Assessment of Dynamic Balance

Each assessment was repeated three times with an interval of 30 seconds between repetitions. Two assessment protocols were used:

1) Sit-to-stand test: The participant was seated on a bench without a backrest or arm support with the knees at 90° flexion and the feet 10 cm apart at the heels. Upon a visual signal (green light) and a verbal command (“stand up”), the participant stood up from the bench and remained motionless, looking at a fixed point at eye height, with the arms relaxed beside the body 12.

2) Stair-climbing test: The participant was placed in an orthostatic position in front of a step that measured 20 cm high. Upon a visual signal (green light) and a verbal command (“climb up”), the participant climbed up the step with the left foot and climbed down the step with the right foot without supporting the right foot on the step; then, the participant climbed down with the left foot parallel to the right foot. After climbing down the step, the participant remained motionless, looking at a fixed point at eye height, with the arms relaxed beside the body. At the end of three measurements, the order of the feet was reversed so that the participant climbed up with the right foot and down with the left foot.

### Statistical Analysis

The data were stored and analysed in SPSS 20.0 for Windows and are presented as averages, medians, and standard deviations. The Mann-Whitney U test was used to compare the quantitative data, and the chi-square test was used to compare qualitative data. A 5% significance level was adopted throughout the statistical analysis.

## RESULTS

### Subject Characteristics

The groups presented no statistically significant differences (Table 1).

### Quality of Life Assessment

The Kendo group showed a statistically significantly higher QoL; on the WHOQOL-bref, these differences were evident in the physical and environment domains, and on the WHOQOL-old, these differences were evident in social participation and in past, present, and future activities (Table 2).

### Functional assessment (muscle strength and dynamic balance)

The groups presented no statistically significant differences in BMI, height, body mass, muscle mass, hand-grip strength, and knee extension and flexion force (Table 3). The Control group was faster in the sit-to-stand test (Table 4).

## DISCUSSION

The martial arts are physical exercises in which each style has its specific benefits, and their contributions to health have been demonstrated in several studies 5,13. However, there is a lack of academic publications regarding the benefits of Kendo. When searching PubMed for the terms “Kendo”, “elderly”, and “quality of life”, no reference to the topic of interest was found.

We assessed a convenience sample of 20 participants and considered the results as a possible pilot for future studies. The main findings of this study were that the practice of Kendo improves the QoL of elderly players, allowing them to remain physically active and maintain good functionality.

The “physical domain” score was higher for the Kendo group and, according to Molzahn et al. 14, is the most important aspect of the assessment. Sampaio and Ito 15 assessed 465 elderly individuals in Japan, and physical activity was demonstrated to have a great influence on the WHOQOL-bref measurement scores. For the WHOQOL 3, physical self-perception depends on how satisfied an individual is with his/her physical and functional condition regardless of the number of comorbidities. Reinforcing this information, the present study demonstrates that superior physical functionality is not enough to guarantee a better QoL because although the Control group scored significantly higher on the functional assessment, the Kendo group scored significantly higher on the WHOQOL evaluation, especially in the physical domain.

The “environment domain” score was higher for the Kendo group. According to Fleck et al. 16, this domain is related to participation and leisure opportunities and to opportunities to acquire new information and skills. Sonati et al. 17 assessed the QoL of 15 kenjutsu players in the city of São Paulo (12 men and three women with an average age of 27.73±7.40 years) and concluded that the physical domain positively influences the psychological domain, which in turn directly influences the environment domain. Pereira et al. 18 and Vagetti et al. 19 reported that the physical domain directly influences the environment domain because an elderly individual with a higher physical self-perception will take more advantage of leisure opportunities and will have better control of his or her environment. This information corroborates the results of the present study because the Kendo group demonstrated statistically significant superiority in the physical domain and, consequently, in the environment domain.

The “past, present, and future activities” facet scores were higher for the Kendo group. According to Power et al. 8, this facet evaluates satisfaction regarding life achievements. This result corroborates the tenets of the Japanese Kendo Federation 5, which advocates “Kendo for life”.

The “social participation” facet score was higher for the Kendo group. According to Power et al. 8, this facet concerns not both activities in the community and everyday activities. This result corroborates the tenets of the Japanese Kendo Federation 5, which posits that contributing to the peace and prosperity of all and serving the country and the society with love are among the values that should be practiced.

We found no statistically significant difference in socio-demographic characteristics, comorbidities, BMI, height, body mass, muscle mass, hand-grip strength, and knee flexor and extensor strength. Tanimoto et al. 20 believe that there is an association between strength and functional capacity when assessing muscle mass, hand-grip strength, and physical performance. Alonso et al. 21 also reported that hand-grip strength correlates with knee flexor and extensor strength to positively affect dynamic balance. Therefore, because there was no significant difference in the strength and muscle mass results, it was expected that there would be no difference in dynamic balance, similar to the findings of Serra et al. 6. However, in our study, when we compared balance in the sit-to-stand test, the Kendo players took longer to perform weight transfer. This difference was significant, but no clinical implications related to balance were observed because the groups were similar in terms of history of falls. This result suggests that the physical activity performed by the Control group was more vigorous than that performed by the Kendo group because according to Pau et al. 22 and Brech et al. 23, vigorous physical activity yields more efficiency when transferring from sitting to standing.

The clinical implication of this study is that those who practice Kendo while ageing improve their QoL. However, the sample size of the two groups is a limitation of the study; therefore, despite being sufficient in terms of statistics (demonstrating a good internal validity), the results must be analysed with caution when applied to the broader population. Future studies with more participants will be performed to identify and describe the benefits of physical activity for the QoL of the elderly.

## AUTHOR CONTRIBUTIONS

de Mendonça DL was responsible for the study design, data collection, data analysis and preparation of the manuscript. Alonso AC was responsible for data collection, data analysis and critical review of the manuscript. Greve JM was responsible for the critical review of the manuscript. Garcez-Leme LE supervised the study and was responsible for the study design and critical review of the manuscript.

## Figures and Tables

**Table 1:** Socio-demographic characteristics and comorbidities of the Kendo and Control groups.

	Kendo N (%)	Control N (%)	χ^2^ (*p*)
**Race or Colour**			
**White**	1 (10%)	2 (20%)	
**Black**	0 (0%)	1 (10%)	1.58 (0.453)
**Yellow**	9 (90%)	7 (70%)	
**Marital Status**			
**Married**	9 (90%)	10 (100%)	1.05 (0.305)
**Widow**	1 (10%)	0 (0%)	
**Education Level**			
**9 to 11 years**	2 (20%)	0 (0%)	
**12 to 16 years**	2 (20%)	4 (40%)	2.66 (0.264)
**>17 years**	6 (60%)	6 (60%)	
**Retired**			
**Yes**	8 (80%)	10 (100%)	2.22 (0.136)
**No**	2 (20%)	0 (0%)	
**Family Income**			
**<1 minimum wages**	1 (10%)	0 (0%)	
**1 to 2 minimum wages**	2 (20%)	1 (10%)	
**2 to 3 minimum wages**	0 (0%)	3 (30%)	
**3 to 5 minimum wages**	2 (20%)	2 (20%)	6.44 (0.168)
**5 to 10 minimum wages**	1 (10%)	3 (30%)	
**10 to 20 minimum wages**	2 (20%)	1 (10%)	
**More than 20 minimum wages**	2 (10%)	0 (0%)	
**Comorbidities**			
**Systemic arterial hypertension**	4 (40%)	6 (60%)	0.80 (0.37)
**Diabetes mellitus**	0 (0%)	2 (20%)	2.22 (0.13)
**Orthopaedic diseases**	2 (20%)	0 (0%)	2.22 (0.13)
**History of previous fractures**	6 (60%)	2 (20%)	3.33 (0.68)
**History of previous falls (≤12 months)**	1 (10%)	3 (30%)	1.25 (0.26)

Chi-square. *p≤0.05.

**Table 2:** Comparison of age, quality of life, WHOQOL-bref domains and facets of the WHOQOL-old between the Kendo and Control groups.

	Kendo Median	Kendo Average (sd)	Control Median	Control Average (sd)	*p*
**Age (years)**	70	71.8 (5.4)	73.50	73.10 (4.8)	0.38
**Quality of life**	4.30	4.39 (0.32)	3.72	3.76 (0.30)	0.002*
**WHOQOL-bref**					
**Physical**	4.21	4.39 (0.32)	3.79	3.64 (0.34)	≤0.0001*
**Psychological**	4.17	4.25 (0.36)	4.08	3.85 (0.59)	0.218
**Social relations**	4.17	4.17 (0.53)	3.83	3.80 (0.55)	0.143
**Environment**	4.25	4.23 (0.36)	3.50	3.53 (0.54)	0.004*
**WHOQOL-old**					
**Sensory abilities**	4.50	4.30 (0.64)	4.25	3.95 (0.71)	0.190
**Autonomy**	4.25	4.00 (0.80)	3.75	3.45 (0.71)	0.063
**Past, present, and future activities**	4.38	4.40 (0.47)	3.88	3.88 (0.32)	0.019*
**Social participation**	4.50	4.43 (0.35)	3.75	3.75 (0.31)	0.001*
**Death and dying**	4.25	4.15 (0.86)	3.50	3.75 (0.91)	0.280
**Intimacy**	4.63	4.45 (0.55)	4.13	4.03 (0.51)	0.089

Mann-Whitney U.

* *p*≤0.05.

Legend: sd-standard deviation.

**Table 3:** Comparison of body mass index, height, body mass, muscle mass, hand-grip strength, and knee extension and flexion force between the Kendo and Control groups.

	Kendo Median	Kendo Average (sd)	Control Median	Control Average (sd)	*p*
**Body Mass Index (kg/m^2^)**	25.20	24.8 (3.3)	25.65	25.34 (3.8)	0.83
**Height (cm)**	165.50	164.5 (5.0)	167.50	166.6 (8.8)	0.57
**Body mass (kg)**	68.20	67.2 (9.0)	64.30	70.70 (15)	0.76
**Muscle mass (kg)**	28.55	28.1 (2.9)	27.00	28.84 (5.7)	0.83
**Hand-Grip Strength**					
**Dominant (kg)**	39.67	40.50 (5.65)	40.99	39.99 (6.61)	0.86
**Non-dominant (kg)**	38.83	39.17 (4.04)	38.00	37.43 (5.74)	0.48
**Knee Extension**					
**TP/BW dominant leg (%)**	181.20	175.59 (44.91)	186.05	179.98 (31.05)	0.72
**TP/BW non-dominant leg (%)**	179.70	182.53 (38.82)	179.10	174.96 (26.01)	0.79
**TW dominant leg (J)**	475.20	449.75 (112.92)	471.35	483.14 (114.91)	0.66
**TW non-dominant leg (J)**	458.55	483.29 (92.51)	483.75	489.02 (123.41)	0.93
**Knee Flexion**					
**TP/BW dominant leg (%)**	105.45	103.8 (26.99)	91.95	94.74 (20.35)	0.48
**TP/BW non-dominant leg (%)**	103.55	99.03 (16.19)	89.20	95.73 (22.73)	0.59
**TW dominant leg (J)**	324.85	310.95 (81.38)	277.9	289.38 (77.37)	0.66
**TW non-dominant leg (J)**	305.60	305.68 (59.80)	306.35	307.00 (74.70)	1.00

Mann-Whitney U *p≤0.05.

Legend: cm – centimetres; kg – kilograms; TP/BW – torque peak adjusted for body weight; TW – total work; N-m – Newton-metres; % – percentage; J – Joules.

**Table 4:** Comparison of balance on the sit-to-stand and stair climbing tests between the Kendo and Control groups.

	Kendo Median	Kendo Average (sd)	Control Median	Control Average (sd)	*p*
**Sit-to-Stand**					
**Transfer time (seconds)**	0.61	0.60 (0.17)	0.39	0.40 (0.16)	0.03*
**Body mass transfer to stand up (%)**	19.50	29.38 (30.34)	20.00	21.80 (6.27)	0.72
**Balance velocity (°/second)**	2.8	2.74 (0.77)	3.6	3.96 (1.12)	0.06
**Stair Climb**					
**Body mass transfer to climb up DS (%)**	38.50	37.88 (9.64)	41.00	41.50 (9.83)	0.53
**Body mass transfer to climb up NDS (%)**	34.50	35.13 (8.36)	39.00	37.80 (13.14)	0.56
**Difference between limbs for climbing up (%)**	8.08	8.18 (14.94)	2.19	19.59 (41.65)	0.79
**Movement time DS (seconds)**	1.78	1.76 (0.29)	1.64	1.71 (0.25)	0.37
**Movement time NDS (seconds)**	1.65	1.68 (0.35)	1.69	1.77 (0.39)	0.72
**Time difference between limbs (%)**	8.06	6.51 (11.94)	-0.62	-1.02 (13.73)	0.29
**Body mass transfer to climb down LND (%)**	39.00	36.63 (11.01)	43.50	45.30 (18.83)	0.27
**Body mass transfer to climb down DS (%)**	45.50	42.13 (12.60)	45.00	49.90 (25.69)	0.79
**Impact difference between limbs while climbing down (%)**	17.39	15.05 (12.66)	4.09	10.20 (37.80)	0.48

Mann-Whitney U

* *p*≤0.05.

Legend: DS – dominant side; NDS – non-dominant side; sd – standard deviation.
